# Femtosecond laser hierarchical surface restructuring for next generation neural interfacing electrodes and microelectrode arrays

**DOI:** 10.1038/s41598-022-18161-4

**Published:** 2022-08-17

**Authors:** Shahram Amini, Wesley Seche, Nicholas May, Hongbin Choi, Pouya Tavousi, Sina Shahbazmohamadi

**Affiliations:** 1 Research and Development, Pulse Technologies Inc., Quakertown, PA 18951 USA; 2 Biomedical Engineering Department, University of Connecticut, Storrs, CT 06269 USA; 3 UConn Tech Park, University of Connecticut, Storrs, CT 06269 USA

**Keywords:** Biomedical engineering, Chemistry, Engineering, Materials science

## Abstract

Long-term implantable neural interfacing devices are able to diagnose, monitor, and treat many cardiac, neurological, retinal and hearing disorders through nerve stimulation, as well as sensing and recording electrical signals to and from neural tissue. To improve specificity, functionality, and performance of these devices, the electrodes and microelectrode arrays—that are the basis of most emerging devices—must be further miniaturized and must possess exceptional electrochemical performance and charge exchange characteristics with neural tissue. In this report, we show for the first time that the electrochemical performance of femtosecond-laser hierarchically-restructured electrodes can be tuned to yield unprecedented performance values that significantly exceed those reported in the literature, e.g. charge storage capacity and specific capacitance were shown to have improved by two orders of magnitude and over 700-fold, respectively, compared to un-restructured electrodes. Additionally, correlation amongst laser parameters, electrochemical performance and surface parameters of the electrodes was established, and while performance metrics exhibit a relatively consistent increasing behavior with laser parameters, surface parameters tend to follow a less predictable trend negating a direct relationship between these surface parameters and performance. To answer the question of what drives such performance and tunability, and whether the widely adopted reasoning of increased surface area and roughening of the electrodes are the key contributors to the observed increase in performance, cross-sectional analysis of the electrodes using focused ion beam shows, for the first time, the existence of subsurface features that may have contributed to the observed electrochemical performance enhancements. This report is the first time that such performance enhancement and tunability are reported for femtosecond-laser hierarchically-restructured electrodes for neural interfacing applications.

## Introduction

**Figure 1:**
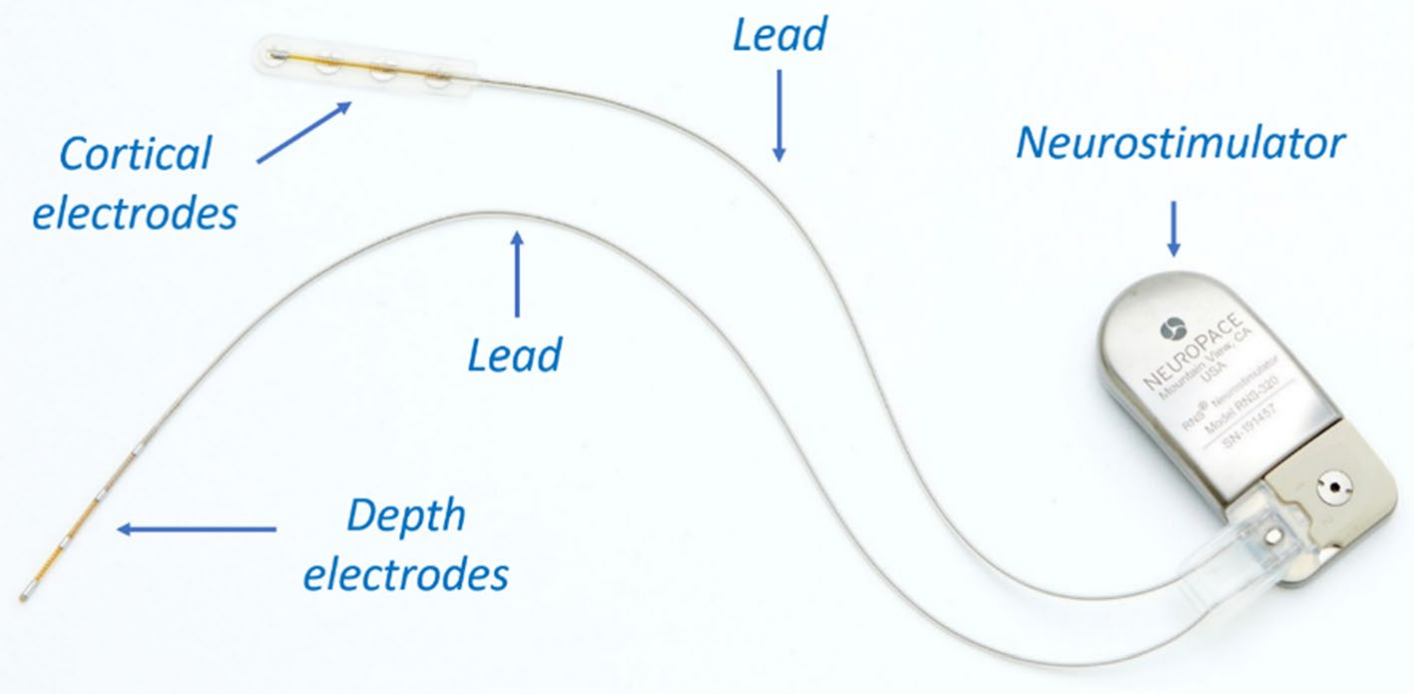
An example of a responsive neurostimulation device (RNS® System, NeuroPace, Inc.; photo used with permission and courtesy of NeuroPace, Inc.); The neurostimulator is implanted in the skull, replacing a similarly shaped section of bone. The cortical strip or depth electrodes (Pt-10Ir alloy) are implanted in or on the epileptic seizure focus.

Ageing population and existence of a multitude of cardiac^1,2^, neurological^3–6^, retinal^7,8^ and hearing disorders,^9,10^ that cannot be cured solely by medication, have resulted in a significant growth in the number of patients who require long-term implantable devices. These devices and their wide range of applications are summarized in Table 1. Implantable devices function via artificial stimulation of the living tissue through transfer of an external electrical signal from a neurostimulator or an implantable pulse generator (IPG) to an implantable electrode or microelectrode array and then across the membrane of the neural cells or tissue^11^. The nervous system is responsible for transportation of the electrical signals that travel from brain to muscles for eliciting muscle motion, and vice versa from the sensory organs to the brain (e.g., sensing, hearing, and vision). If a nerve is injured and the communication between the brain and the periphery is disrupted, as in the case of spinal cord injury for example^12–15^, it is possible to use a device to either restore the function that the brain cannot control^4^ or record this information from the nervous system. Over the past several decades, many patients across the globe have been reliant on implantable devices for life-critical and life-sustaining functions^16–18^, which has resulted in massive transformations in these devices. Particularly, there has been a strong trend towards device miniaturization since smaller implantable devices are desired to make them compatible with normal human activities and enhance the comfort of the host^19,20^. Therefore, all components of such devices need to be optimized for weight, size, and patients’ comfort. The majority of these devices consist of three major components: (1) neurostimulator or IPG, which contains a battery and electronics; (2) electrodes or microelectrode arrays, responsible for sensing and recording intrinsic neurological or cardiac activity and also delivering pulses for pacing and stimulating purposes; and (3) leads, which bridge between the IPG and the electrodes or (micro)electrode arrays^1,3,6,8,20,21^. Figure 1 shows an example of a neurostimulation device and all three major components outline above.

**Table 1:** Long-term implantable devices and their applications.

Device	Application
Cardiac rhythm management devices^1,2,21–24^	To treat and manage arrhythmia-related diseases for either too slow of a heart rhythm (e.g. through implantation of a pacemaker) or too fast of a heart rhythm (e.g. through implantation of a defibrillator)
Cochlear implants^9,25–27^	To treat hearing disorders for patients with severe to profound hearing loss by providing acoustic input through electrical stimulation of the auditory nerve
Retinal prosthesis and bionic vision^20,26,28–34^	To restore some vision to patients who have become blind from degenerative retinal diseases such as retinitis pigmentosa and age-related macular degeneration
Neurostimulation devices^20,35–45^ for: Spinal cord stimulation^3,12,14,15,46–49^ Sacral nerve stimulation^50–55^ Vagal nerve stimulation^56–61^ Deep brain stimulation^62^ Responsive neurostimulation^63^	Pain management^3,15,64–66^ Treating symptoms of Parkinson's disease Treating tremor and severe psychiatric disorders such as depression and obsessive–compulsive disorders^60,67–70^ Suppressing and treating epileptic seizures and diagnosing epilepsy^61,63,71,72^ Recording electrical signals from the surface of the brain also known as electrocorticography (ECoG)^6,73–75^ Artificial limb control^74,76^ Suppression of involuntary movements^70^ Partial restoration of motor skills for those who have lost functionality through loss of limb or spinal cord injury^13,77,78^ Lost body function replacement^32^ Blood pressure control and modulation^59^ Development of brain computer interfaces^6,79,80^

**Figure 2:**
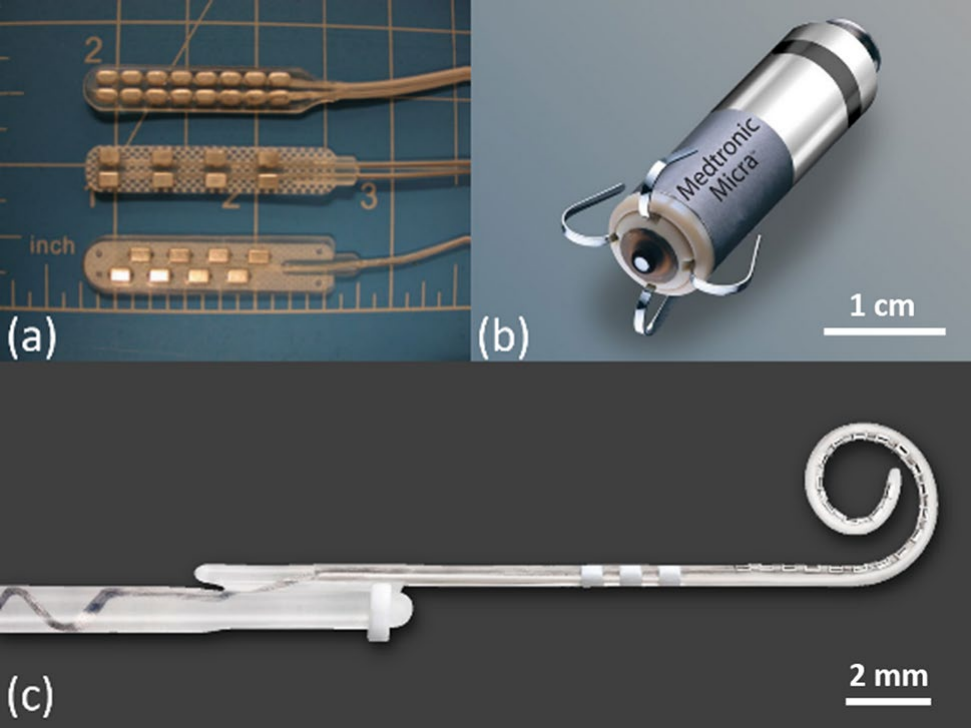
(**a**) Typical two-column spinal cord stimulation paddle electrode arrays with 8 and 16 electrodes (photo reproduced with permission from Bradley, K. *Pain Medicine* 7, 2006^1^); (**b**) a leadless pacemaker that is implanted directly into the heart where the anode is a circumferential ring located in the proximal portion of the device and responsible for cardiac pacing (photo with permission and courtesy of Medtronic); (**c**) a cochlear implant electrode array with 22 electrodes (photo with permission and courtesy of Cochlear Limited, Sydney, Australia).

Electrodes and microelectrode arrays are the basis of many emerging devices. They are fabricated to have very specific sizes, geometries, profiles, as well as electrical, electrochemical, and mechanical properties to match the biological requirements of their intended applications. Electrodes are vastly diverse because different applications demand different electrode types in terms of their size, invasiveness, selectivity, material composition and performance^3,4,9,32,81,82^. A microelectrode array is a very thin piece of plastic carrying embedded metallic structures, which is implanted into the human body to interact with the nervous system^3,4,9,32,81–84^. A higher density array of electrodes allows a greater number of discrete neurons or groups of neurons to be activated which results in increased localization and control of the desired biological response^11,62,85^. However, manufacturing limitations have hindered progress in the development of high-density microelectrode arrays^86,87^. In most implantable devices, high-performing electrodes or microelectrode arrays are characterized by low impedance (for sensing and recording purposes), high charge injection capacity (for safe and reversible stimulation purposes), and high capacitance for cardiac pacing applications^42,88,89^. We will use these parameters as performance metrics throughout this report. Figure 2 shows some examples of state-of-the-art electrodes and electrode arrays currently in practice.

Considering the overall dimensions of the implant, fabrication of electrodes that are small enough for communication with neurons is technologically feasible^81^. However, a size reduction of the actual conducting site is inevitably accompanied by an increase in the impedance of the electrode, and consequently a decrease in signal to noise ratios. Therefore, the size of an electrode for clinical use is determined by a trade-off between high selectivity (obtained by small size) and optimized electrochemical characteristics^81^. Larger electrodes have greater Geometric Surface Area (GSA), and thus can inject more charge before exceeding the electrochemically safe limits^42^. However, their large size limits the spatial selectivity and resolution of the device^90^. To increase the charge injection capacity, for delivering a higher resolution signal and improving performance^86,90^, one can increase GSA by increasing the number of electrodes. Nevertheless, considering the space limitations within organs such as brain, spinal cord, cochlea, and eye, such increase in number of electrodes must be accompanied by a reduction in electrode size, which significantly reduces the amount of charge that can be delivered. This adversely impacts the device performance and defeats the purpose of increasing the number of electrodes. To overcome the aforementioned tradeoff, an alternative approach is to increase the number of electrodes to achieve high selectivity, where each electrode has a small GSA, but an enhanced Electrochemical Surface Area (ESA)^42,86,90^, to achieve high charge transfer capability and low impedance. By maximizing ESA, while minimizing GSA, a large number of electrodes can be accommodated in the device, promoting enhanced performance, selectivity, fidelity, and lower power consumption. Increasing ESA has been achieved through two classes of techniques: (1) surface technologies through which a different material (e.g*.*, coatings, thin films, and nanomaterials with greater electrochemical performance than the electrode itself) is added or deposited onto the electrode surface; (2) physical and electrochemical techniques to enhance/alter surface roughness of the electrode. Typical electrode coatings include but are not limited to iridium oxide thin films (IrO_2_)^39,42,44,91–105^, titanium nitride coatings (TiN)^38,96,106,107^, black or porous platinum (Pt) coatings^81,108–110^, conductive polymers^83,86,111–116^, two-dimensional materials^117,118^, carbon nanotubes^119–122^, and nanostructured scaffolds^123^. Despite their ability to enhance electrochemical performance of electrodes, some of these coating material platforms: (1) pose technological challenges in the manufacturing environment, such as not lending themselves to serial or in-line processing, the need for costly time-consuming vacuum and batch processes, and the need for use of masks to selectively coat areas of interest on the electrode surface; (2) have some unfavorable properties, such as poor adhesion of the coatings and additive layers to the underlying electrode surface and shortcomings associated with long-term durability. Examples of such challenges are provided in Table 2.

**Table 2:** Coating Techniques to increase ESA and their associated challenge.

Coating and thin films	Technological challenges and drawbacks
Pt black or (nano) porous Pt	Loss of material, and therefore surface area, due to abrasion^124,125^
Iridium oxide (IrO_2_)	Poor adhesion to the underlying substrates, low robustness and susceptibility to delamination under prolonged stimulation and poor long-term durability^126–128^
Conducting polymers (e.g.*,* PEDOT)	Structural defects such as cracking and delamination^129–131^, leading to further detachment of the coating, thus affecting the functionality of the electrode

In light of the described shortcomings of coating and thin film approaches, commercially viable technologies that can increase the electrochemical performance of electrodes and microelectrode arrays, while negating the need for coating or depositing a new material onto the surface, are deemed valuable. Such aim can be achieved using surface treatment techniques, which include: (1) electrochemical roughening^132,133^; (2) physical methods that use a laser to alter surface morphology via etching, melting, or roughening of the electrode surface^28,134^. Laser restructuring of neural interfacing electrodes and microelectrode arrays to improve their electrochemical performance has been studied in the literature in an ad hoc fashion^28,85,134,135^. Table 3 summarizes these studies.

**Table 3:** Summary of studies reported in the literature utilizing laser technologies to fabricate and improve electrode performance in neural interfacing applications.

Publication	Findings
Schuettler et al.^75,128,136^	Reported on the use of laser cutting and laser patterning for fabrication of platinum electrodes
Schuettler^134^	Reported on the use of laser surface melting with ~ 4.5 times increase in the surface area of their electrodes
Green et al*. *^85,137^	Found that the surface achieved by melt processing, imparted from the relatively long pulse duration required for roughening, increased surface bound oxides of Pt, preventing the full electrode area from being utilized for charge transfer
Stover et al.^138^	Investigated the feasibility and the potential use of femtosecond lasers to create defined channels into a conventional cochlear implant electrode array to allow for fluid-based drug delivery
Dodds et al.^28^	Reported on the use of laser patterning to fabricate microelectrode arrays for a stimulating retinal prosthesis with improved surface area and electrochemical activity
Henle et al.^73^	Reported on the first long term *in-vivo* study of implanted micro-ECoG electrodes manufactured and roughened by laser technology
Green et al.^137^	Reported that the surface area of an electrode was increased by ~ 2.5 times using an Nd:YAG laser with nanosecond pulse widths to roughen the electrode surface; they also reported that the safe charge injection limit was increased by ~ 3.5 times
Green et al.^90^	Reported on fabrication of electrode arrays with various laser patterning and roughening techniques with improvements in electrochemical performance and lower impedance compared to untreated surfaces of equal dimensions
Zhang et al*.*^139^	Investigated performance of laser-patterned platinum electrodes, in particular laser interference patterning, for use in visual prosthesis systems

**Figure 3:**
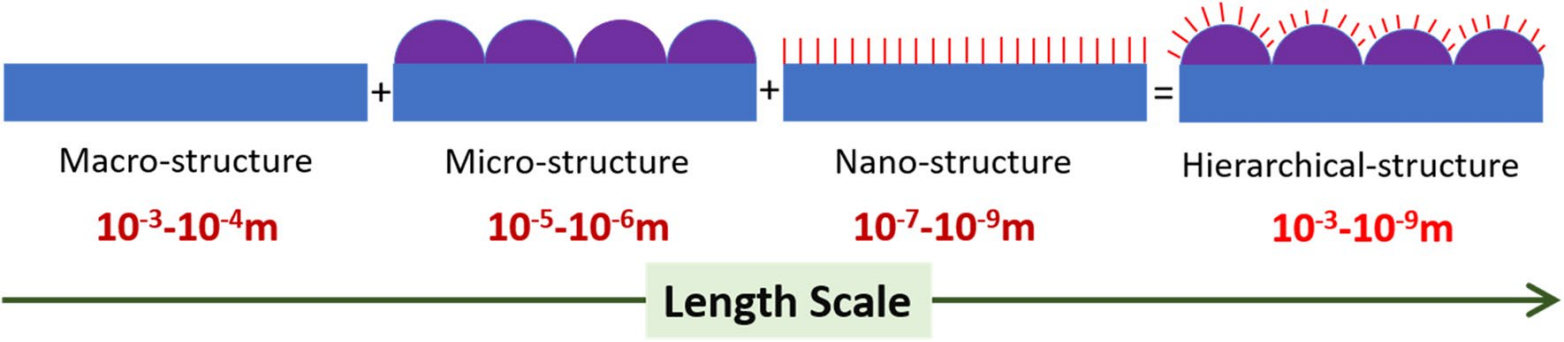
Schematic of a hierarchically structured surface consisting of topographic features spanning a variety of length scales. For most applications, these varying length scales are the coarse-scale rough structures (~ 1–100 µm) and a finer structure subset (~ 5–100 nm) on top of the coarse structures.

The concept of hierarchical surfaces and structures have been vastly studied in the literature. Many natural and man-made materials exhibit bulk or surface structures on more than one length scale, meaning that the structural or surface elements themselves have a structure within. In neural interfacing applications, thin films of iridium oxide (IrO_2_), palladium oxide (PdO), ruthenium oxide (RuO_2_), rhodium oxide (Rh_2_O_3_), and their binary solid solution thin films^103–105^, for example, exhibit a hierarchical (also known as *fractal*, in this context) surface structure when synthesized under specific processing parameters. This hierarchy plays a large part in achieving an ultra-high ESA that renders them ideal thin film materials for neural interfacing applications. It is hypothesized here that formation of hierarchical surface structures on electrodes and microelectrode arrays, i.e. electrodes with topographic surface features comprised of varying length scales as illustrated in the schematic of Fig. 3 can give rise to electrochemical performance because the surface properties shall be governed by both the chemical composition of the electrode surface and the morphological effect of nanostructures within the micrometer-scale areas of the hierarchical surface^140–148^.

Several methods have been reported in the literature for the fabrication of hierarchical surface structures on different materials^146^ such as spin-coating^149^, polymer imprinting^150–152^, self-assembly^153^, replica casting of natural surfaces^143^, nanolithography^141,144,154,155^, chemical etching^148^, and nanoparticle deposition^142,156^. The possibility of material nano-processing, using femtosecond laser pulse ablation, was first reported by Pronko et al*.*^157^ in 1995 and others^158–165^ thereafter. Because of its ultrashort pulse duration and the large laser peak fluence, this method allows restructuring of almost all classes of materials with the desired precision and without the occurrence of noticeable heat affected zones^166^. Several studies specifically have reported on the use of femtosecond lasers for hierarchical and surface nanostructuring of various materials^146,158,166–173^. The developed techniques for surface nanostructuring using femtosecond laser include mask projection^174^, near-field ablation^175^, laser-assisted chemical etching^176^, nanotexturing by deposition from a femtosecond laser ablation plume^177^, nanostructuring of thin metal films by femtosecond laser induced melt^178^, plasmonic nanoablation^179^, and interferometric femtosecond laser ablation^180,181^. Importantly, use of femtosecond lasers for fabrication of biomimetic surfaces has gained significant attention in recent decades^166,182^.

## Objectives

In this report, we have investigated the applicability and performance benefits of femtosecond laser hierarchical surface restructuring. We then explored the tunability of performance as a function of two of the most important and readily accessible laser parameters, i.e*.,* fluence and average power. In addition to establishing the correlation between laser parameters and performance, we sought to understand what contributed to such unprecedented performance in these hierarchically restructured electrodes. The widely adopted reasoning in the literature has been the increased surface area and/or surface roughening. Using correlative confocal microscopy (CM) and scanning electron microscopy (SEM), the restructured electrodes were fully characterized in all three dimensions, i.e.*,* 2D lateral texture and morphology with SEM and height information with CM with nanometer resolution. While there exists some loose correlation between surface parameters and performance, we show that surface parameters alone fail to fully explain the trend and the extent of enhanced electrochemical performance. Further cross-sectional analysis using high resolution focused ion beam (FIB) cross-sectioning and subsequent SEM imaging show, for the first time, the existence of subsurface features that may have contributed to the observed electrochemical performance and calls for further studies that investigate both surface and subsurface features.

## Materials and methods

### Femtosecond laser hierarchical surface restructuring

Electrodes or microelectrode arrays have specific electrochemical performance requirements for their intended applications. Therefore, the ability to selectively tune their performance metrics by tweaking tunable laser parameters is of great interest for both researchers and medical device manufacturers. Of the several laser processing parameters that enable surface tunability, *Average Power* and *Fluence* are the focus of this study as they can be readily tuned in almost all commercial lasers. A series of flat 0.3 mm thick Pt-10Ir electrodes were hierarchically restructured in two experiments. In experiment 1, Average Power was varied from 0.6 to 3.35 W, while all the other known/controllable lasering parameters were kept constant. In experiment 2, Fluence was varied from 12.3 to 2 J/cm^2^, while Average Power was kept constant at about 17 W. Table 4 outlines the Average Power and Fluence values that were used in these two experiments. Additionally, and to demonstrate feasibility and practicality of hierarchical surface restructuring in real world applications, a series of Pt-10Ir electrodes with flat and 3D/complex geometries were restructured.

**Table 4:** Laser processing parameters (Average Power and Fluence) used in experiments 1 and 2.

Average power (W) Experiment 1	0.61	1.07	1.52	1.98	2.44	2.90	3.35
Fluence (J/cm^2^) Experiment 2	12.30	6.15	4.10	3.07	2.46	2.05	–

The laser system used was a diode pumped Yb:YAG solid state laser (Coherent StarFemto, Santa Clara, CA) that generates 300 fs pulses with a central wavelength of 1030 nm. The rationale behind using a femtosecond laser in this work was that literature clearly shows femtosecond lasers can be utilized for material processing with minimal to zero undesired collateral damage (e.g., due to dissipation of generated heat)^183,184^. Such ability is key for achieving a surface restructuring process that is controllable and repeatable, with no undesired artifacts. The experiments were performed in air, under ambient conditions. Surface patterns were created via a graphical editor (Visual Laser Marker provided by Coherent), tied into the axis controls, and the beam path was directed using a deflection head. Electrodes were mounted onto a vacuum plate mounted on a tip-tilt stage (Edmunds Optics, Barrington, NJ) on an XYZ-translation stage. The electrodes were leveled to within 5 µm delta across the surface using an optical non-contact displacement transducer (Micro Epsilon, Ortenburg, Germany). Electrodes were brought directly under the deflection head to minimize incident angle.

### Confocal and scanning electron microscopy and FIB cross-sectioning

Restructured electrodes underwent correlative confocal microscopy (CM) and scanning electron microscopy (SEM) imaging. CM was performed in a ZEISS Smart proof 5 (ZEISS, Jena, Germany) and Keyence VK 3000 (Keyence, Osaka, Japan). The SEM and FIB/SEM imaging were performed using a ZEISS Crossbeam 340 (ZEISS, Oberkochen, Germany). SEM Imaging was conducted with a secondary electron detector at an accelerating voltage of 10 kV under various magnifications. Magnifications of 50k, 20k, 10k, 5k, 2k, 1k, and 500 corresponding to pixel sizes of 2.23, 5.58, 11.16, 22.33, 55.82, 111.6, and 223.3 nm were used, which enabled the investigation of the hierarchal structures at various length scales. Further, micrographs were taken at 0° and 45° tilt angles, enabling better visualization of the overall topology of the surface and correlation with the 3D confocal data. In order to reveal subsurface features induced by restructuring, FIB cross-sectioning was performed using a gallium FIB at a current of 100 nA and an accelerating voltage of 30 kV to create a trench with dimensions of 50 µm width and 100 µm length and 80 µm depth. Polishing of the cross-section was done in multiple steps, using lower currents down to 1 nA to ensure best cross-sectional wall surface quality. The confocal data provides nanometer height resolution (the exact resolution depends on the selected objective) while SEM provides similar resolution in lateral directions. The correlation between the two, enabled by Mountain Software (Digital Surf, Besancon, France), allows for full characterization of surfaces in all three dimensions. The objective lens and acquisition modes were chosen such that the surface roughness of the restructured electrodes could be fully captured with the highest resolution possible. Due to the difficulty associated with aligning features such as peaks and valleys from one image to another, 180 µm × 180 µm areas that contain 25 full peaks, 20 half peaks and four quarter peaks were extracted from every image that was acquired. The following image processing steps were applied to obtain various surface parameters: (1) Filling non-measured points (i.e. filling points on the surface where no confocal information was present using interpolation; the total number of non-measured points for our images were less than 5%); (2) Removing outliers; (3) Leveling; (4) Filling non-measured points (only if the previous process yielded any additional non-measured points (which is always less than 1%); (5) Thresholding to remove foreign objects; (6) Extracting surface parameters. Surface parameters established by the ISO 25178 standard were calculated. Root mean square (RMS) height of the surface (S_q_), and the surface area ratio (S_dr_) were selected as two parameters that reflect “surface roughness” and “added surface area” also reported by Taylor et al*.* for similar neural interfacing applications^104^. Their mathematical formulation is provided in Eqs. (1) and (2):

*RMS roughness* (*S*_*q*_)

1 $${S}_{q}=\sqrt{\frac{1}{A}\underset{A}{\overset{}{\iint }}{Z}^{2}\left(x,y\right)dxdy}$$

*Surface area ratio *(*S*_*dr*_)

2 $${S}_{dr}=\frac{1}{A}\left[\underset{A}{\overset{}{\iint }}\left(\sqrt{\left[1+{\left(\frac{\partial z(x,y)}{\partial x}\right)}^{2}+{\left(\frac{\partial z(x,y)}{\partial y}\right)}^{2}\right]}-1\right)\right]dxdy$$

where Z denotes the height of each point on the surface and A is the area of the sample. RMS roughness corresponds to the standard deviation of the height distribution and is a widely used parameter due to its robustness being less sensitive to measurement noise. The surface point cloud can be triangulated for surface area measurements. In particular, S_dr_ calculates the area of each individual triangular and sums them up to define the curvilinear area that follows each asperity and texture element of the surface. This area is then divided by the horizontal area in order to determine how much the surface differs from a horizontal plane. Throughout this report, we use S_dr_ to quantitatively characterize added surface area.

### Electrochemical measurements

**Figure 4:**
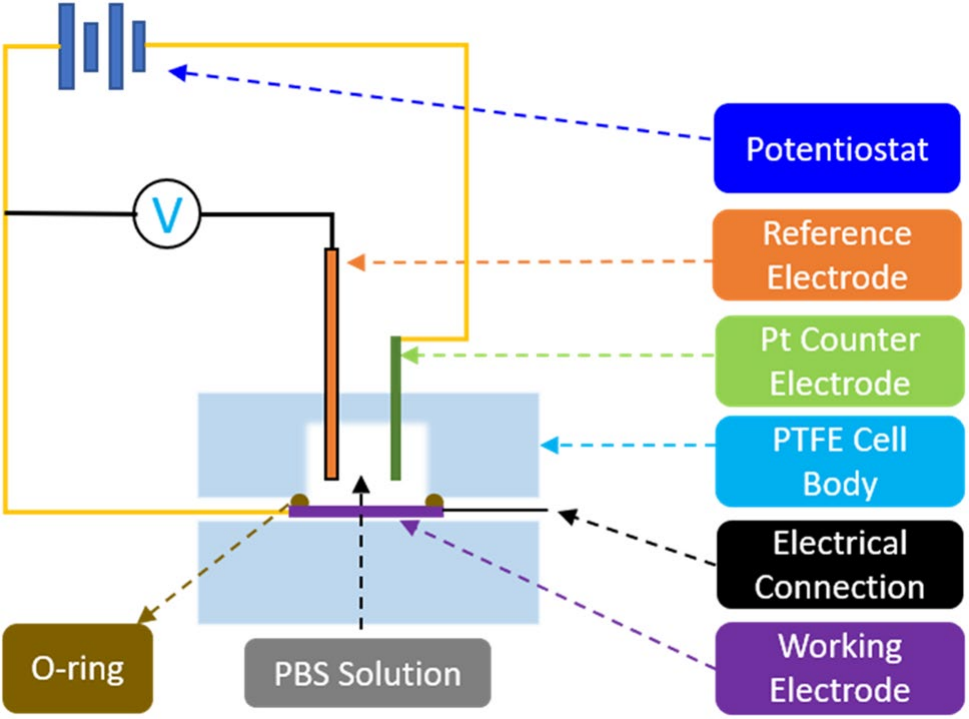
Schematic of the test setup used for CV and EIS measurements.

Charge storage capacity (CSC) is an important property to consider when determining the usefulness of an electrode or a microelectrode array^42,90^ and can be measured via cyclic voltammetry (CV). The voltage in a CV test is restricted to a range where no detrimental electrochemical reactions occur to the biological tissue or nerves. Since tissue reactions are application specific, in practice, these voltage limits are commonly determined by the so called “water window”, representing the potential-range where oxidation or reduction currents will not lead to formation of hydrogen or oxygen at the electrode/tissue interface (e.g. −0.6 V to 0.8 V vs. a Ag/AgCl reference electrode)^42^. In this work, CV was used to measure CSC and electrochemical impedance spectroscopy (EIS) was used to measure impedance and specific capacitance. Both CV and EIS tests were performed in a three-electrode Teflon® plate cell (Fig. 4), comprising an Ag/AgCl reference electrode (ALS-Co Ltd., RE-1B, Tokyo, Japan), a coiled Pt counter-electrode, and hierarchically restructured electrodes as the working electrodes. The geometric surface area (GSA) of the working electrodes in the cell was 0.09 cm^2^. The electrolyte used was a commercially available phosphate-buffered saline (PBS) solution (Blood Bank Saline, Azer Scientific, Morgantown, PA). All potentials were recorded with respect to the Ag/AgCl reference electrode. All CV tests were performed at room temperature and at a 50 mV/s voltage sweep rate (ν) between potential limits of −0.6 V and 0.8 V, beginning at open-circuit potential (OCP) and sweeping in the positive direction first. As outlined earlier, potential windows were selected to ensure water electrolysis did not occur. EIS measurements were performed at OCP and measured over a frequency range of 0.1–10^5^ Hz using a 10 mV root-mean-square (V_rms_) sinusoidal excitation voltage amplitude about a fixed potential between −0.6 V and 0.8 V. All CV and EIS measurements were performed using a Gamry potentiostat (5000E interface, Warminster, PA) and the vendor supplied software. All data reported for CV and EIS are an average of three electrodes per condition, tested three times, *i.e.,* a total of 9 measurements. Specific capacitance was calculated using EIS data and common Randles model.

## Results and discussion

### Hierarchical surface structures

**Figure 5:**
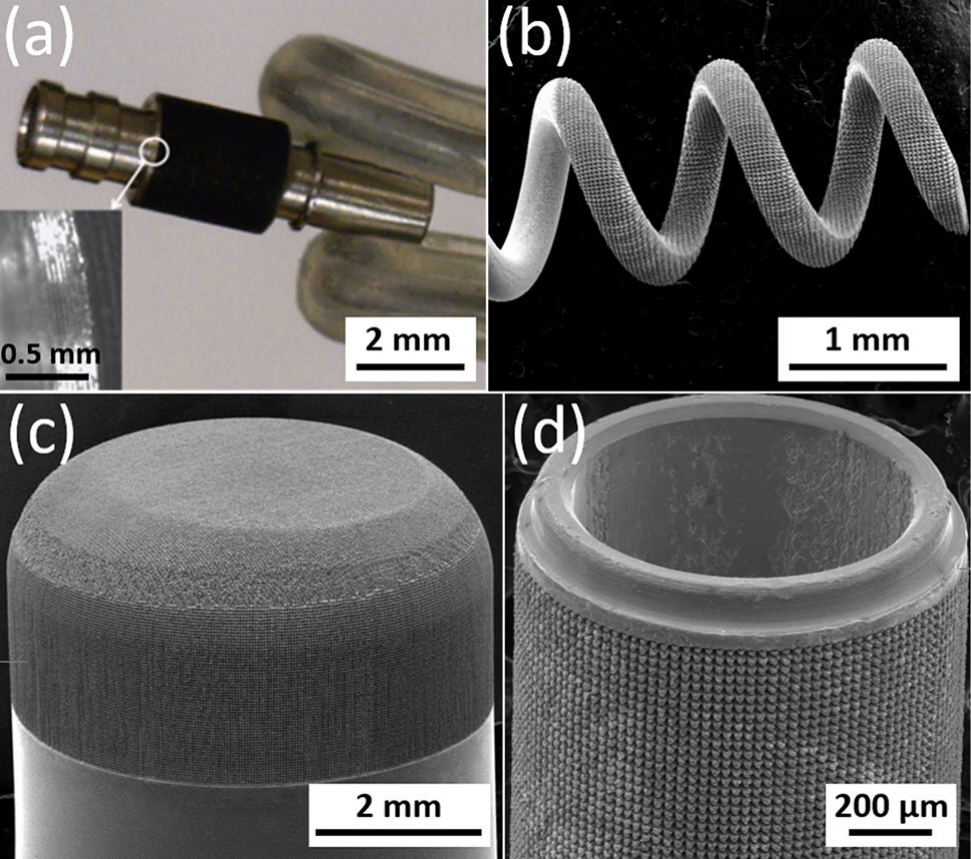
SEM micrographs of hierarchically restructured electrodes for various neural interfacing applications: (**a**) a cylindrical, and, (**b**) a helix Pt-10Ir electrode for cardiac rhythm management applications; (**c**) a rivet-style Pt-10Ir electrode for an electrophysiology mapping catheter and, (**d**) a cylindrical Pt-10Ir electrode for use in a percutaneous spinal cord stimulation electrode array.

**Figure 6:**
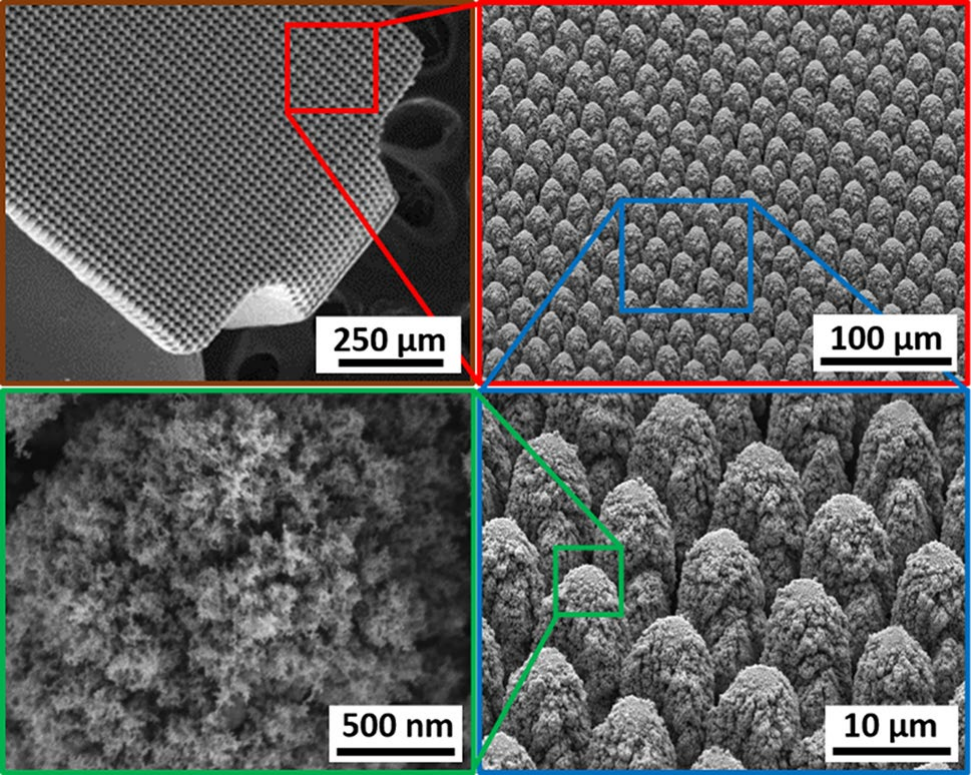
SEM micrographs of the hierarchical surface structure induced on the surface of a Pt-10Ir alloy electrode used for a paddle-lead spinal cord stimulation electrode array.

As shown in the optical and SEM micrographs of Fig. 5, hierarchical surface restructuring was employed successfully as a robust surface modification technology on a diverse range of electrode shapes and geometries for various neural interfacing applications, e.g. cylindrical (Fig. 5a) and helical (Fig. 5b) Pt-10Ir electrodes for cardiac rhythm applications, rivet-style Pt-10Ir electrodes for ultrahigh density mapping electrophysiology catheters (Fig. 5c) and a cylindrical (Fig. 5d) Pt-10Ir electrode for use in percutaneous spinal cord stimulation electrode arrays. The hierarchical surface structure created as a result of restructuring can be observed in the SEM micrographs of a flat Pt-10Ir electrode targeted for use in a paddle-lead spinal cord stimulation electrode array (Fig. 6). The micrographs reveal that the surface hierarchy is notable by a periodic topography comprised of coarse-scale mound-like features that are several microns wide and ~ 10 to 20 µm high and a finer structure subset on top of the mound-like structures in the range of about a few nanometers to a few hundred nanometers in size. The observed pattern in Fig. 6 has been maintained throughout this study for preserving the larger scale structures. This allows for the investigation of tunability independent of the pattern focusing on the most commonly accessible laser parameters of fluence and average power. The authors acknowledge the need for exploring pattern (*i.e.,* the geometric path of laser spot on the surface) as a tunable parameter in future studies but believe lessons learned from the current work provides valuable insights toward such studies and can confine an otherwise prohibitively broad experimental field.

**Figure 7:**
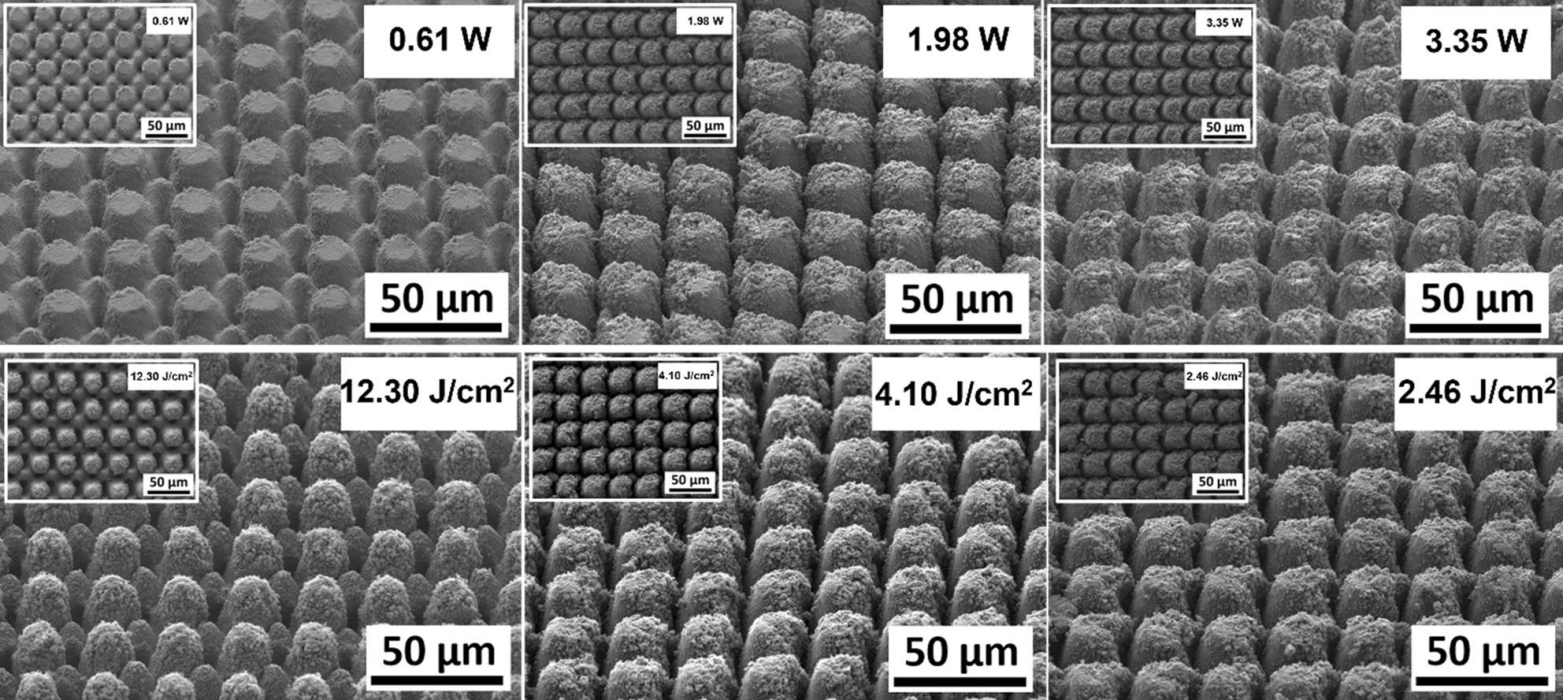
Representative scanning electron microscope (SEM) micrographs of hierarchically restructured surfaces of Pt-10Ir electrodes at 0.61, 1.98, and 3.35 W (top row) average power and 12.30, 4.10, and 2.46 J/cm^2^ (bottom row) fluence.

**Figure 8:**
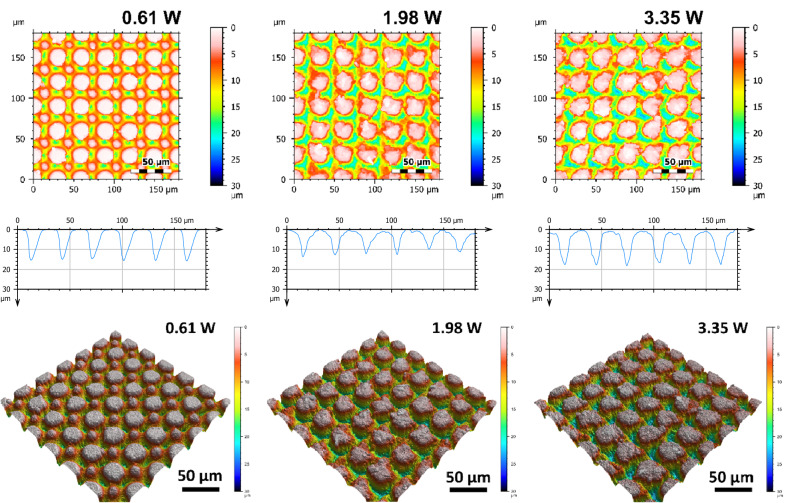
Representative 2D (top row), 1D (middle row), and 3D (bottom row) confocal maps of hierarchically restructured surfaces of Pt-10Ir electrodes at 0.61, 1.98, and 3.35 W Average Power. Note that the cross-sectional profile (1D confocal map) is obtained at the center of the map along the horizontal direction.

**Figure 9:**
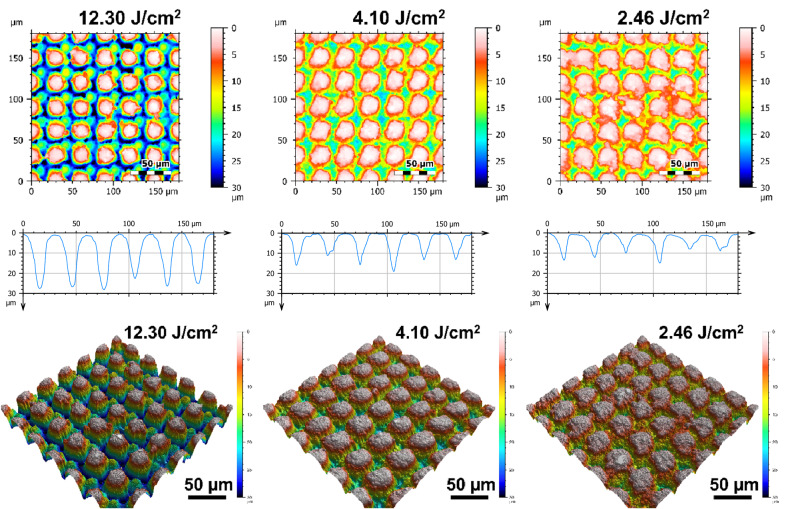
Representative 2D (top row), 1D (middle row), and 3D (bottom row) confocal maps of hierarchically restructured surfaces of Pt-10Ir electrodes at 12.30, 4.10, and 2.46 J/cm^2^ Fluence. Note that the cross-sectional profile (1D confocal map) is obtained at the center of the map along horizontal direction.

**Figure 10:**
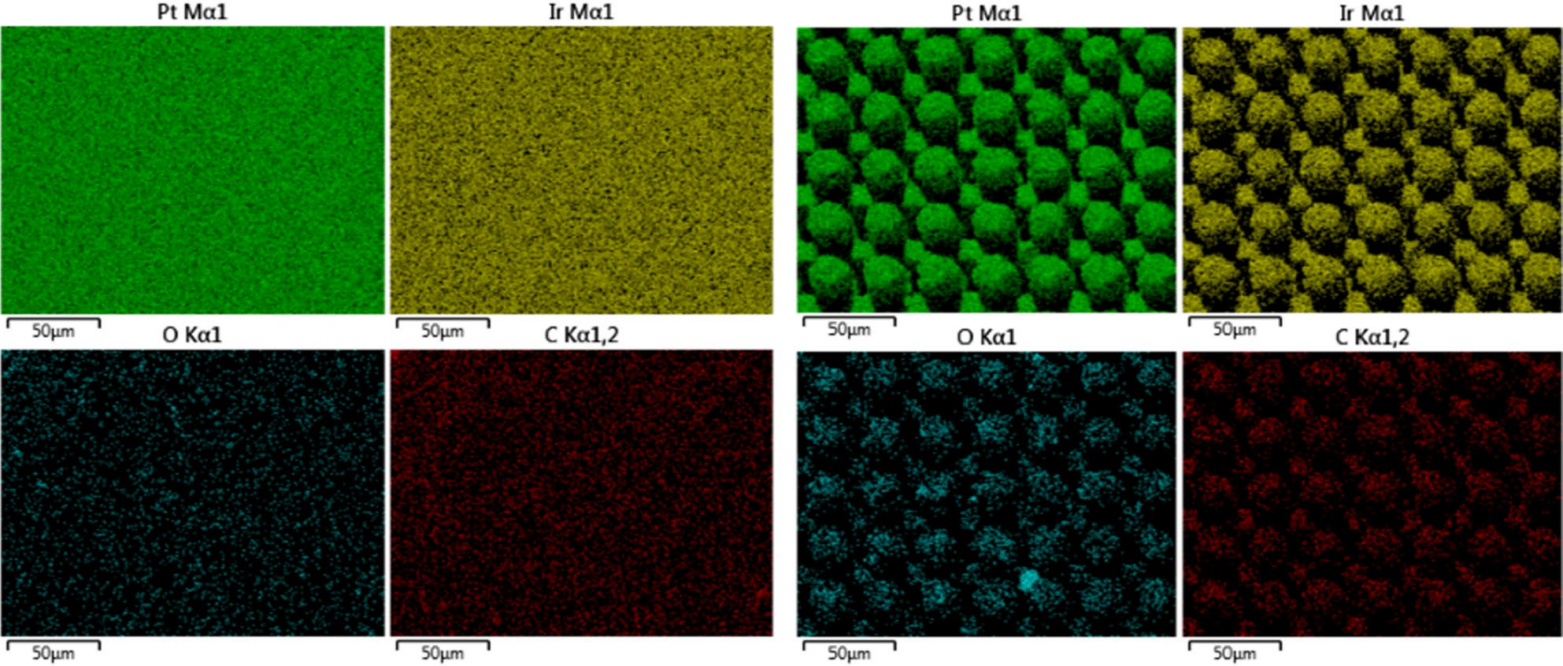
Elemental EDS maps of (left) an un-restructured Pt-10Ir electrode, and (right) a hierarchically restructured Pt-10Ir electrode processed at 12.30 J/cm^2^ fluence.

Figure 7 shows representative SEM micrographs of the electrodes from experiment 1 restructured at 0.61, 1.98, and 3.35 Watts (top row) and electrodes from experiment 2 restructured at 12.3, 4.1, and 2.46 J/cm^2^ of fluence (bottom row). All SEM micrographs were taken at 45° tilt angles, enabling better visualization of the overall topology of the surface, while the insets were captured at 0° tilt angles. These SEM micrographs qualitatively show that the prevalence of smaller-length-scale features on the electrode surface is higher at higher average power. This observation is quantitatively confirmed by correlated confocal images provided in Fig. 8, showing representative 2D heat-map view (top row) and 3D view (bottom row) confocal maps of hierarchically restructured Pt-10Ir electrodes at 0.61, 1.98, and 3.35 W average power. Representative confocal images for 12.30, 4.10, and 2.46 J/cm^2^ fluence are provided in Fig. 9. Similarly, increase in roughness and texture as a function of average power is observed in SEM and confocal images. The 2D heat map view confocal images and SEM micrographs show that the increased roughness presents itself as increased undulations. In images with variable average power the increased roughness is accompanied with increased depth of structures while varying fluence does not present a similar trend. The deviation from circular shape is also more pronounced in Fig. 9 (fluence) than Fig. 8 (average power), significantly reducing the gaps between peaks. Energy dispersive spectroscopy (EDS) was performed at 0° tilt angles for both an un-restructured and hierarchically restructured Pt-10Ir electrodes. Figure 10 shows the compositional EDS maps of both electrode surfaces. Table 5 summarizes the elemental composition of both electrode surfaces. Note that oxygen concentration increased by ~ 5% after hierarchical surface restructuring at 12.30 J/cm^2^ fluence.

**Table 5:** Compositional analysis of the two electrodes from Fig. 10 (i.e., an un-restructured Pt-10Ir electrode and a hierarchically restructured Pt-10Ir electrode processed at 12.30 J/cm^2^ fluence.

Element	Composition (wt.%) (un-restructured Pt-10Ir electrode)	Composition (wt.%) (hierarchically restructured Pt-10Ir electrode at 12.30 J/cm^2^)
Pt	85.36	78.81
Ir	5.77	6.30
C	7.70	8.88
O	1.17	6.01

### Correlation of laser parameters and electrochemical performance and the role of surface properties

**Figure 11:**
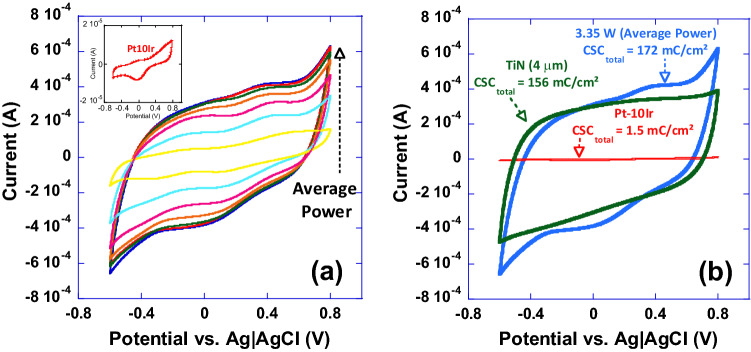
Cyclic voltammograms of, a) a series of electrodes restructured under varying restructuring conditions (0.61 to 3.35 W) and a pristine un-restructured Pt-10Ir electrode (inset), and, b) a 4 µm thick TiN coating for comparison with an electrode restructured at 3.35 W, and a pristine Pt-10Ir electrode; noteworthy is the two orders of magnitude increase in CSC_total_ for the electrode restructured at 3.35 W average power compared to its un-restructured counterpart.

In order to investigate the performance behavior of the observed surfaces, cyclic voltammograms of all electrodes from experiment 1 restructured while varying average power and a pristine un-restructured Pt-10Ir electrode (inset) are shown in Fig. 11a. Total Charge Storage Capacity (CSC_total_) was calculated according to Eq. (3) by integrating the area under the cyclic voltammograms:

3 $$CSC= \frac{1}{\nu GSA}\underset{{V}_{1}}{\overset{{V}_{2}}{\int }}i\left(V\right)dV$$

Here, it is clearly observed that tunability can be achieved by varying average power. Increase in average power is shown to continuously enhance CSC_total_ (Fig. 11a). To provide a better context and for the sake of comparison, cyclic voltammograms of a 4 µm thick TiN coating, an electrode restructured at 3.35 W, and a pristine Pt-10Ir electrode are shown in Fig. 11b. Electrodes restructured at 3.35 W not only show over two orders of magnitude increase in their CSC_total_ compared to their un-restructured Pt-10Ir counterparts, but also their CSC_total_ exceeds that of the 4 µm thick TiN coating routinely used in cardiac rhythm management applications_._ This is the first time that such performance enhancement and tunability are reported for laser restructured electrodes. The pristine Pt10Ir electrode exhibits distinct oxidation and reduction peaks similar to Pt electrodes^42^. The laser restructured Pt10Ir electrodes, on the other hand, exhibit substantially larger voltammograms that are both semi-rectangular, indicating double-layer capacitance similar to TiN, and also contain an oxidation peak at 0.8 V and a small reduction peak near 0.1 V inherent to Pt-10Ir, as shown in the inset CV voltammogram of the pristine Pt-10Ir electrode.

**Figure 12:**
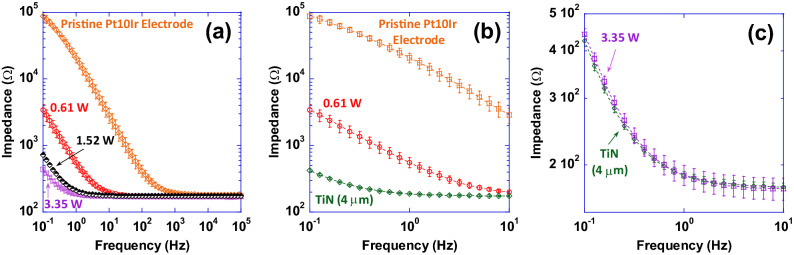
(**a**) Impedance magnitude as a function of frequency (plotted in the 0.1–10^5^ Hz frequency range) for a pristine unrestructured Pt-10Ir electrode, and select hierarchically restructured electrodes as a function of average power (only those electrodes restructured at 0.61, 1.52 and 3.35 W are shown for simplicity), (**b**) impedance magnitude as a function of frequency (plotted in the 0.1–10 Hz frequency range) for a 4 µm thick TiN coated Pt-10Ir electrode and a pristine Pt-10Ir electrode for comparison with the restructured electrode at 0.61 W, and, (**c**) impedance magnitude as a function of frequency (plotted in the 0.1–10 Hz frequency range) for the 4 µm thick TiN coated Pt-10Ir electrode and the Pt-10Ir electrode restructured at 3.35 W.

**Figure 13:**
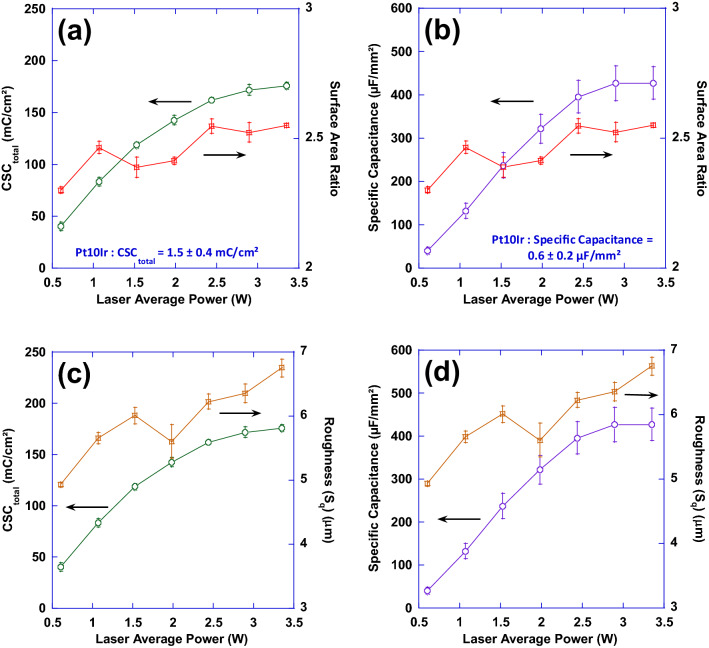
Plots of surface area ratio (S_dr_) as well as total charge storage capacity (**a**) and specific capacitance (**b**) as a function of laser average power; Also, plots of mean surface roughness (S_q_) as well as total charge storage capacity (**c**) and specific capacitance (**d**) as a function of laser average power.

Impedance magnitude as a function of frequency (plotted in the 0.1–10^5^ Hz frequency range) for select electrodes from experiment 1, the pristine Pt-10Ir electrode and the TiN coating are shown in Fig. 12. Most notably, at frequencies below 1000 Hz, EIS tests and impedance measurements (Fig. 12) exhibit approximately up to two orders of magnitude reduction in impedance for hierarchically restructured electrodes (at 3.35 W) compared to the pristine Pt-10Ir counterparts. At higher frequencies, all electrodes exhibit resistive behavior dominated by electrolyte conductivity. Most notably, as illustrated in Fig. 12c, the impedance behavior of the electrode restructured at 3.35 W is nominally identical to that of the TiN coated electrode. More notably, the EIS measurements and capacitance calculations demonstrate over 700-fold increase in specific capacitance (Fig. 13) after hierarchical surface restructuring (at 3.35 W). Additionally, hierarchical surface restructuring has the unique advantage and possibility of engineering the electrodes’ ESA through variation and tunability of laser parameters. Here, such tunability is conveniently achieved by simply dialing a different average power value for the laser. Similar trends are observed for fluence. However, there still remains the question of what drives such performance and tunability and whether the widely adopted reasoning of increased surface area and roughening of the electrodes are the key contributors to the observed increase in performance.

### The relationship between laser parameters, surface structure and performance

**Figure 14:**
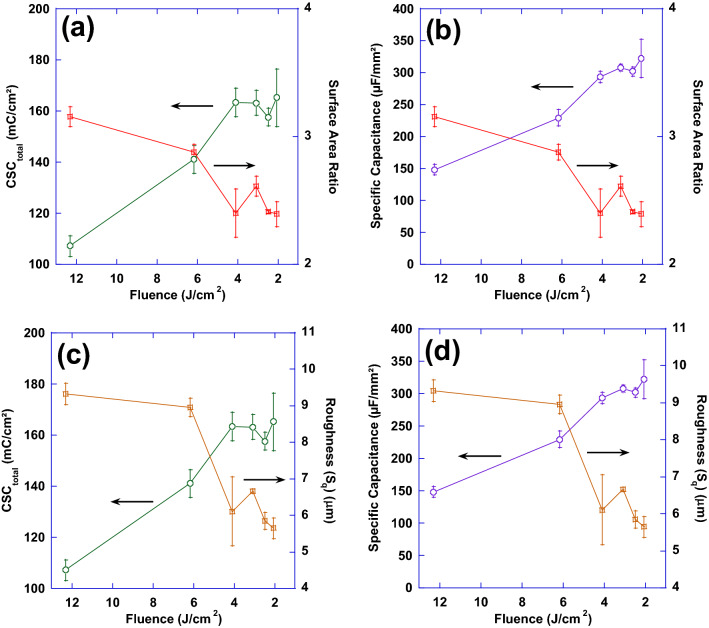
Plots of surface area ratio as well as total charge storage capacity (**a**) and specific capacitance (**b**) as a function of laser fluence; also, plots of mean surface roughness (S_q_) as well as total charge storage capacity (**c**) and specific capacitance (**d**) as a function of laser fluence.

Figures 13 and 14 show the relationship between the variations of laser processing parameters (average power and fluence), surface parameters and performance. In Fig. 13, performance metrics (CSC_total_ and specific capacitance) and surface parameters (S_dr_ and S_q_) have been correlated with average power while Fig. 14 demonstrates that correlation with fluence. While both performance metrics of CSC_total_ and specific capacitance exhibit a relatively consistent increasing behavior with average power and fluence, surface parameters tend to follow a less predictable behavior negating a direct relationship between these surface parameters and performance. It is evident from Fig. 13 that an increase in performance—when average power is increased—does not necessarily guarantee a consistently increasing trend in either of the surface parameters. Additionally, Fig. 14 shows that despite the relatively consistent increasing trend in performance—when fluence increases—both surface parameters exhibit a decreasing trend.

**Figure 15:**
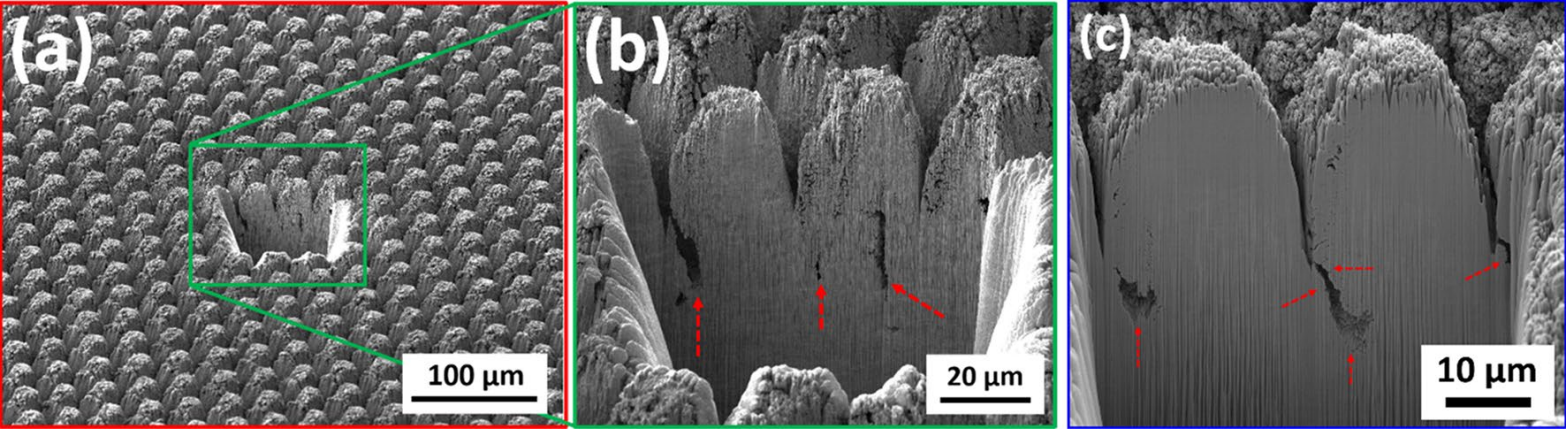
Focused ion beam (FIB) cross sections of hierarchically restructured Pt-10Ir alloy electrodes restructured at 4.10 J/cm^2^ fluence (**a,b**) and 1.98 W average power (**c**); red dashed arrows show subsurface features likely attributed to laser shock waves.

These observations and trends can be attributed to several potential contributors. Firstly, looking at Figs. 8 and 9, one can clearly see that the prevalence of finer nanoscale features with higher frequencies is more pronounced on electrodes with improved performance. The lower performance surfaces have almost ideal circular shape while surfaces with higher performance deviate from circularity and the vicinity of peaks exhibit high frequency undulations. However, when measured quantitatively, other features of surfaces might have dominated the calculations. In our opinion, this calls for devising new functional surface parameters different from conventional surface metrics that can better correlate the surfaces to the performance metrics observed. Another key neglected factor is the existence of any subsurface structures that might be invisible to surface sensitive imaging techniques such as the ones used in this study, i.e. SEM and CM. The subsurface features herein refer to the structural changes that are made to the electrodes below the visible surface. These include voids, cracks, fissures, and the like features. It is hypothesized that these subsurface features—not visible using the presented surface characterization techniques but whose structure is connected to the surface terrains—can contribute to the overall performance metrics of the sample and play an important role in increasing the accessible electrochemical surface area of the electrodes. To explore this further, FIB cross-sections of several electrodes restructured in this study were obtained to investigate whether features invisible to imaging techniques utilized herein exist, or not. Figure 15 shows representative FIB cross-sections of two electrodes hierarchically restructured at 4.10 J/cm^2^ fluence (Fig. 15a,b) and 1.98 W average power (Fig. 15c). Subsurface features (shown by red dashed arrows in Fig. 15) that are consistently occurring close to the valleys are evident in both FIB cross sections of Fig. 15. The existence of such features can likely be attributed to laser shock waves that induce void further away from the spot location of lasers. Although femtosecond lasers are perceived to have significantly less heat and induced shock waves than their counterparts, they still do exist and can create subsurface features as evidenced here.

## Conclusions

In this work, a novel method was introduced for hierarchical surface restructuring of electrode surfaces, using femtosecond laser technology, to promote tunability and controllability of their electrochemical performance for a wide range of neural interfacing applications. The performance of a series of hierarchically restructured electrodes was evaluated and compared with those of un-restructured electrodes as well as TiN coated electrodes and the advantages of laser restructured electrodes over the other two were discussed. Further, tunability of performance metrics, via variation of lasering parameters, was shown and the role of surface and subsurface parameters was investigated. It was demonstrated that surface RMS and added surface area are not able to fully describe the trends observed in performance metrics; thus, further studies are required to correlate surface parameters with performance metrics more confidently. Finally, we have shown that even in the presence of femtosecond pulses, there are potential shock wave induced structures below the surface and away from the interaction spot between the laser and the electrodes. Subsurface features can also contribute to performance. Future studies that include both surface and cross-sectional characterization can better correlate the effect of subsurface features and performance. Lastly, we acknowledge the need for exploring laser patterning as a tunable parameter in future studies but believe lessons learned from the current work provides valuable insights toward such studies and can confine an otherwise prohibitively broad experimental field.

## Supplementary Information

Supplementary Information.

## Data Availability

All data generated or analyzed during this study are included in this published article [and its supplementary information files].
